# Structural insights into the nucleotide base specificity of P2X receptors

**DOI:** 10.1038/srep45208

**Published:** 2017-03-23

**Authors:** Go Kasuya, Yuichiro Fujiwara, Hisao Tsukamoto, Satoshi Morinaga, Satoshi Ryu, Kazushige Touhara, Ryuichiro Ishitani, Yuji Furutani, Motoyuki Hattori, Osamu Nureki

**Affiliations:** 1 Department of Biological Sciences, Graduate School of Science, The University of Tokyo, 2-11-16 Yayoi, Bunkyo-ku, Tokyo, 113-0032, Japan; 2 Integrative Physiology, Department of Physiology, Graduate School of Medicine, Osaka University, 2-2 Yamada-oka, Suita, Osaka, 565-0871, Japan; 3 Institute for Molecular Science, 38 Nishigo-Naka, Myodaiji, Okazaki, 444-8585, Japan; 4 Department of Applied Biological Chemistry, Graduate School of Agricultural and Life Sciences, The University of Tokyo, 1-1-1 Yayoi, Bunkyo-ku, Tokyo, 113-8657, Japan; 5 ERATO Touhara Chemosensory Signal Project, JST, The University of Tokyo, 1-1-1 Yayoi, Bunkyo-ku, Tokyo, 113-8657, Japan; 6 State Key Laboratory of Genetic Engineering, Collaborative Innovation Center of Genetics and Development, Department of Physiology and Biophysics, School of Life Sciences, Fudan University, 2005 Songhu Road, Yangpu District, Shanghai, 200438, China

## Abstract

P2X receptors are trimeric ATP-gated cation channels involved in diverse physiological processes, ranging from muscle contraction to nociception. Despite the recent structure determination of the ATP-bound P2X receptors, the molecular mechanism of the nucleotide base specificity has remained elusive. Here, we present the crystal structure of zebrafish P2X4 in complex with a weak affinity agonist, CTP, together with structure-based electrophysiological and spectroscopic analyses. The CTP-bound structure revealed a hydrogen bond, between the cytosine base and the side chain of the basic residue in the agonist binding site, which mediates the weak but significant affinity for CTP. The cytosine base is further recognized by two main chain atoms, as in the ATP-bound structure, but their bond lengths seem to be extended in the CTP-bound structure, also possibly contributing to the weaker affinity for CTP over ATP. This work provides the structural insights for the nucleotide base specificity of P2X receptors.

Adenosine 5-triphosphate (ATP), an important intracellular energy source, is also an extracellular neurotransmitter that controls cell functions via cell surface receptors^1^,^2^. P2X receptors are trimeric cation channels that permeate a wide range of monovalent and divalent cations, by responding to extracellular ATP^3^,^4^,^5^. In the P2X receptors, each subunit has two transmembrane helices, connected by a relatively large extracellular domain. Seven subtypes of P2X receptors, named P2X1 to P2X7, are widely expressed in excitable and non-excitable cells, and are involved in diverse physiological processes such as muscle contraction, nociception, taste signal transduction and macrophage activation^6^,^7^. Accordingly, P2X receptors have attracted great interest as potential drug targets for the treatment of epilepsy, neurodegenerative disorders, and chronic inflammation^8^,^9^,^10^. The previously determined crystal structures of zebrafish P2X4 (zfP2X4)^11^,^12^, tick P2X (amP2X)^13^ and human P2X3 (hP2X3)^14^ in the presence or absence of ATP revealed the overall architecture of the receptor and provided structural insights into the molecular mechanisms of ATP binding and ATP-dependent gating cycle. More recently, the crystal structures of the panda P2X7 (pdP2X7) receptor provided the molecular basis of the subtype-specific non-competitive antagonist recognition in P2X7 receptors^15^.

While P2X receptors lack affinities for GTP and UTP, some P2X receptors have weak affinity for CTP (20 to 50 of times lower affinity than for ATP)^16^,^17^,^18^. The ATP-bound zebrafish P2X4 structure, and the superimposition of other nucleoside triphosphates onto its ATP binding site, suggested a possible model for the nucleotide base specificity of P2X receptors^12^. According to the proposed model based on the ATP-bound structure, a conserved threonine residue within the agonist binding site forms a hydrogen bond with the adenine ring of ATP, but not with the cytosine ring of CTP, and the difference in the number of hydrogen bonds between each nucleotide base and P2X receptors (three for ATP, two for CTP) is responsible for the weaker affinity for CTP over ATP^12^. However, the mechanism of the nucleotide base specificity of P2X receptors remains elusive, due to the lack of structural information for the P2X receptor in complex with other nucleoside triphosphates, particularly the low-affinity pyrimidine agonist, CTP. In addition, the mechanism of the pyrimidine base recognition by P2X receptors is quite interesting from the viewpoint of structure-based drug design, because some pyrimidine derivatives act as P2X antagonists^19^,^20^.

In this study, we determined the crystal structure of zebrafish P2X4 in complex with CTP, and conducted structure-based electrophysiological and spectroscopic analyses to reveal the mechanism underlying the nucleotide base specificity of P2X receptors.

## Results

### The nucleotide base specificity of zebrafish P2X4

**Figure 1:**
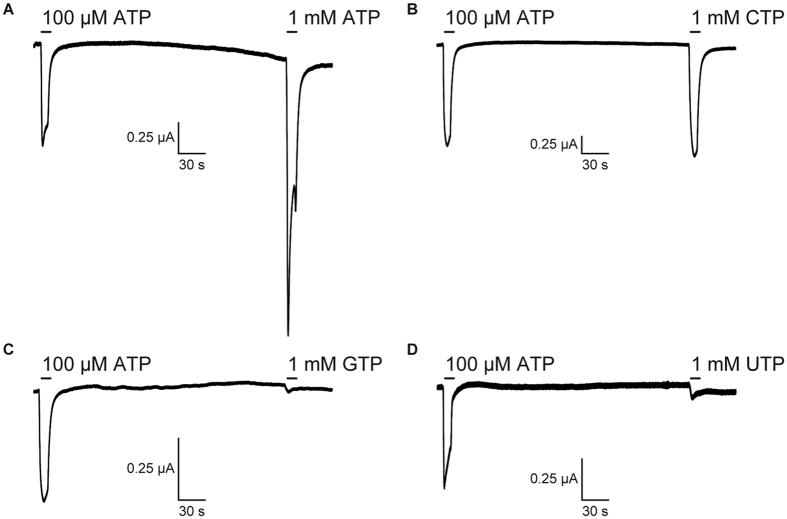
Current responses of zebrafish P2X4 evoked by nucleoside triphosphates. (**A**–**D**) Representative currents of zebrafish P2X4 WT, evoked first by 100 μM ATP and then by (**A**) 1 mM ATP, (**B**) 1 mM CTP, (**C**) 1 mM GTP and (**D**) 1 mM UTP. Each nucleotide was applied for 10 sec. The holding potential was −70 mV.

To assess the nucleotide base specificity of zebrafish P2X4 (zfP2X4), we first performed Two Electrode Voltage Clamp (TEVC) recording of *Xenopus* oocytes. As shown previously^21^, the initial “priming” application with a low concentration of ATP increases P2X receptors sensitivity to second or more applications of ligands, which is useful for evaluating the affinity of ligands. Therefore, we expressed zfP2X4 in oocytes and recorded the zfP2X4-associated currents evoked by the application of high concentrations (1 mM) of nucleoside triphosphates (ATP, CTP, GTP and UTP), following the prior application of 100 μM ATP. We detected specific ATP- and CTP-evoked currents (Fig. 1A,B), but not GTP- and UTP-evoked currents (Fig. 1C,D), consistent with the previous electrophysiological analyses of P2X receptors^16^,^17^,^18^,^22^,^23^,^24^,^25^,^26^.

### Structure of the CTP-bound zfP2X4

**Figure 2:**
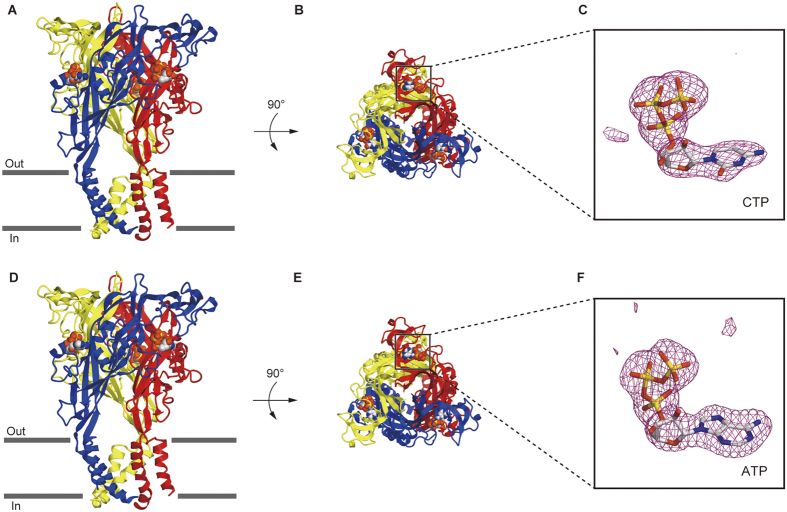
Overall comparisons of the CTP-bound and ATP-bound ΔP2X_4_-C structures. (**A**,**B**) The CTP-bound ΔP2X_4_-C structure viewed parallel to the membrane (**A**) and from the extracellular side (**B**). (**C**) The omit *F*_*o*_ − *F*_*c*_ map contoured at 4σ, showing the electron density of CTP. CTP is depicted by a stick model. (**D**,**E**) The ATP-bound ΔP2X_4_-C structure viewed parallel to the membrane (**D**) and from the extracellular side (**E**). (**F**) The omit *F*_*o*_ − *F*_*c*_ map contoured at 4σ, showing the electron density of ATP. ATP is depicted by a stick model.

To investigate the CTP recognition mechanism, we crystallized zebrafish P2X4 constructs (ΔP2X_4_-C) in the presence of 3 mM CTP (about 7,600-fold excess over the IC_50_ (ref. 12)). We collected X-ray diffraction data at 2.8 Å resolution, and determined the structure (Fig. 2). The subunit of CTP-bound structure (Fig. 2A,B) is quite consistent with that of the previously determined ATP-bound structure (Fig. 2D,E), with root mean square deviation (RMSD) values below 0.4 Å for 324 Cα atoms (Supplementary Fig. S1B), indicating that CTP can trigger the conformational changes similar to those by ATP.

**Figure 3:**
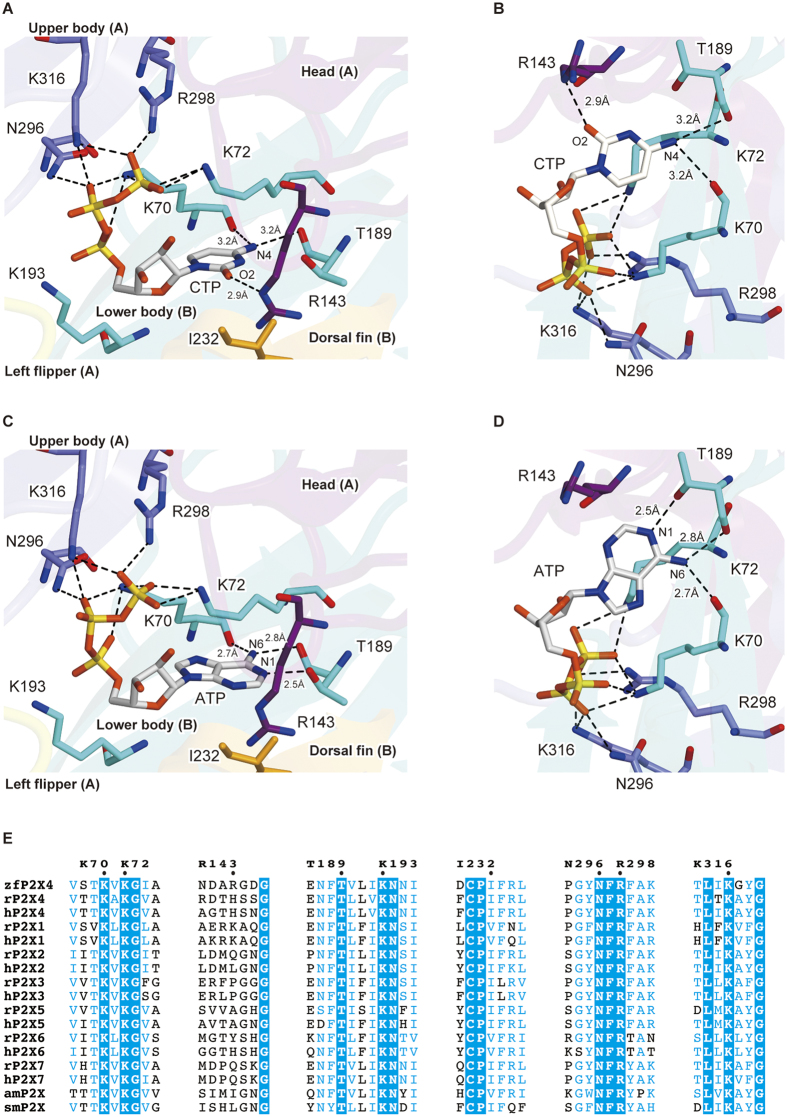
Close-up views of the CTP and ATP binding sites. (**A**–**D**) Close-up views of the CTP binding site in the CTP-bound ΔP2X_4_-C structure (**A**,**B**), and the ATP binding site in the ATP-bound ΔP2X_4_-C structure (**C**,**D**). Side chains of amino acid residues and nucleoside triphosphates are depicted by stick models. The molecule is colored according to the previously proposed dolphin-like model. Each dotted black line and number indicates a hydrogen bond and its length (<3.3 Å) respectively. (**E**) Sequence alignment around agonist binding site of P2X receptors. Amino acid sequences were aligned using Clustal Omega (https://www.ebi.ac.uk/Tools/msa/clustalo/) and are shown using ESPript3 (http://espript.ibcp.fr/ESPript/ESPript/). For the sequence alignment, zebrafish P2X4 (zfP2X4, GI: 12656589) and the following P2X receptors were used: human (hP2X1, 4505545; hP2X2, 25092719; hP2X3, 28416925; hP2X4, 116242696; hP2X5, 209572778; hP2X6, 6469324; and hP2X7, 29294631), rat (rP2X1, 1352689; rP2X2, 18093098; rP2X3, 1030065; rP2X4, 1161345; rP2X5, 1279659; rP2X6, 1279661; and rP2X7, 1322005), Gulf Coast tick (amP2X, GI: 346469461), and blood fluke (smP2X, 51988420).

The overall structure forms a chalice-like shape assembled into a homotrimeric architecture, with a large extracellular domain and a small transmembrane domain, that is consistent with the previously determined P2X structures^11^,^12^,^13^,^14^,^15^. Each subunit has two transmembrane helices and resembles the shape of a dolphin^11^ (Supplementary Fig. S1A). We observed a strong residual electron density for the nucleoside triphosphate at the agonist binding site (Fig. 2C). The phosphate groups adopt a unique bent conformation and are directly recognized by the side chains of N296, R298, K316 from one subunit, and K70 and K72 from the adjacent subunit (Fig. 3A,B), consistent with the ATP-bound structure (Fig. 3C,D). The shape of the electron density for the base group was smaller than that in the ATP-bound structure (Fig. 2C,F), and could be readily assigned as the cytosine of the CTP molecule.

### Cytosine base recognition

The CTP-bound structure revealed the mechanism of cytosine base recognition by the receptor (Fig. 3). While the side chain of the conserved threonine, T189, forms a hydrogen bond with the adenine ring in the ATP-bound structure (Fig. 3C,D), there is no equivalent hydrogen bond between the cytosine ring and the side chain of T189 in the CTP-bound structure (Fig. 3A,B). Instead, we identified another hydrogen bond between the cytosine base and the receptor. The O atom of the cytosine interacts with the side chain of R143 (Fig. 3A,B), which had not been predicted in the previous superimposed model based on the ATP-bound structure^12^. Furthermore, the NH_2_ atom of cytosine is recognized by the main chain carbonyl oxygen atoms of K70 and T189 (Fig. 3A,B), as similarly observed in the ATP-bound structure (Fig. 3C,D), but their bond lengths seem to be 0.4–0.5 Å longer in the CTP-bound structure (Fig. 3B,D). Although it is difficult to conclude whether these bonds are really extended or not at this resolution of 2.8 Å, it should be noted that the residues constituting the agonist binding site are well ordered with lower B-factors and clear electron density in both the ATP-bound and CTP-bound structures (Supplementary Fig. S2).

Overall, the structural comparison between the CTP-bound and ATP-bound structures suggested that both CTP and ATP form three hydrogen bonds between their nucleotide bases and the receptor, but employ different residues (Fig. 3). This result totally differs from the previously proposed model by superposing CTP onto ATP in the ATP-bound structure, in which the cytosine ring of CTP was predicted to form only two hydrogen bonds with the receptor^12^.

### Mutation assay of the amino acid residues at the agonist binding site by Electrophysiology

**Figure 4:**
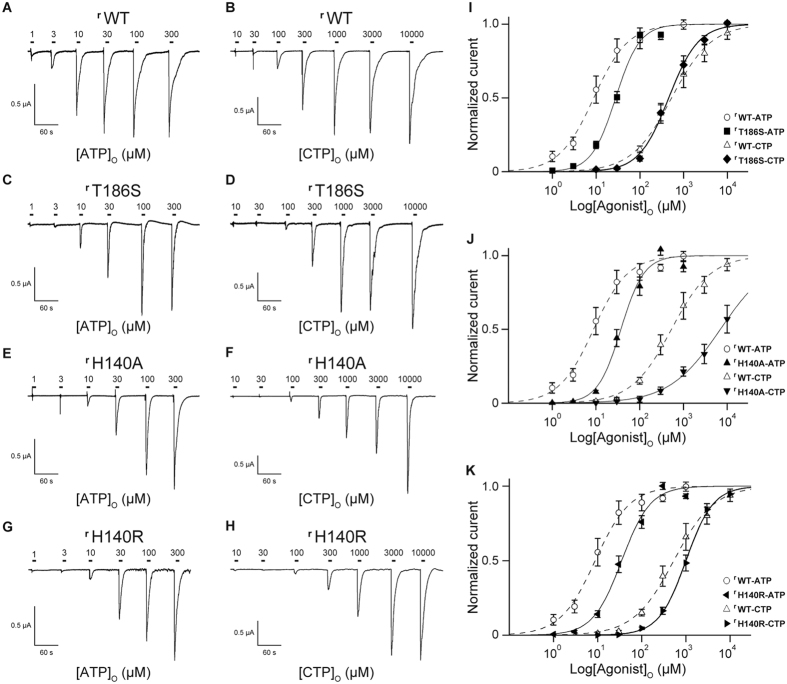
Current responses of ^r^WT, ^r^T186S, ^r^H140A and ^r^H140R evoked by ATP and CTP. (**A**,**B**) Representative currents of ^r^WT evoked by ATP (1–300 μM) (**A**) and CTP (10–10,000 μM) (**B**). (**C**,**D**) Representative currents of the ^r^T186S mutant evoked by ATP (1–300 μM) (**C**) and CTP (10–10,000 μM) (**D**). (**E**,**F**) Representative currents of the ^r^H140A mutant evoked by ATP (1–300 μM) (**E**) and CTP (10–10,000 μM) (**F**). (**G**,**H**) Representative currents of the ^r^H140R mutant evoked by ATP (1–300 μM) (**G**) and CTP (10–10,000 μM) (**H**). (**I**–**K**) Concentration-response curves evoked by ATP and CTP in the ^r^WT and the ^r^T186S mutant (**I**), in the ^r^WT and the ^r^H140A mutant (**J**) and in the ^r^WT and the ^r^H140R mutant (**K**). Symbols are defined in the figure, and bars depict means ± SEM (n = 7–9).

To further investigate the functional roles of the residues involved in the base recognition, T189 and R143, we performed an electrophysiological analysis with the rat P2X4, since its electrophysiological properties have been well established^27^,^28^,^29^,^30^. We first assessed the functional role of T189 in zfP2X4 and introduced mutations at the equivalent residue of the rat P2X4 to create three mutants (^**r**^T186A, ^**r**^T186V and ^**r**^T186S; the superscript “r” means rat P2X4). While ATP- and CTP-evoked currents were detected from oocytes expressing the ^**r**^WT and the ^**r**^T186S mutant, no ATP- and CTP-evoked currents were detected from oocytes expressing the ^**r**^T186A and ^**r**^T186V mutants (Supplementary Fig. S3), indicating the hydrophilic environment provided by the side chain of amino acid residue at this position might be important for the channel activity. Therefore, we examined the ^**r**^WT and the ^**r**^T186S mutant. We analyzed the dose-response relationships to ATP and CTP in the ^**r**^WT and the ^**r**^T186S mutant, while no GTP- and UTP-evoked currents were detected from the ^**r**^WT and the ^**r**^T186S mutant (Supplementary Fig. S4A,B). The ^**r**^T186S mutant exhibited reduced ATP affinity (EC_50_ = 29.9 ± 3.1 μM, n = 9) (Fig. 4C,I) as compared with ^**r**^WT (EC_50_ = 16.2 ± 5.4 μM, n = 9) (Fig. 4A,I), whereas the ^**r**^T186S mutant exhibited similar CTP affinity (EC_50_ = 557 ± 120 μM, n = 9) (Fig. 4D,I) as compared with ^**r**^WT (EC_50_ = 564 ± 170 μM, n = 9) (Fig. 4B,I). These results are consistent with the ATP-bound and CTP-bound structures in which the side chain of ^r^T186 forms a hydrogen bond with the adenine ring, but not with the cytosine ring (Fig. 3).

Next we assessed the functional role of R143 in zfP2X4, as its side chain forms a hydrogen bond with the cytosine ring in the CTP-bound structure (Fig. 3A,B). This arginine residue is substituted with a similar basic residue, histidine (^r^H140), in rat P2X4 (Fig. 3E). Since the side chain of arginine is also suggested to interact with the O atom of the cytosine, we created and tested the corresponding mutants (^r^H140A and ^r^H140R) of ^r^H140. We analyzed the dose-response relationships to ATP and CTP in the ^**r**^WT and the ^**r**^H140 mutants, while no GTP- and UTP-evoked currents were detected from the ^**r**^WT and the ^**r**^H140 mutants (Supplementary Fig. S4A,C,D). The ^**r**^H140A mutant exhibited about 2.5-fold reduced ATP affinity (EC_50_ = 42.8 ± 6.3 μM, n = 8) (Fig. 4E,J) and 14-fold reduced CTP affinity (EC_50_ = 7,990 ± 1,500 μM, n = 7) (Fig. 4F,J) as compared with ^**r**^WT while the ^**r**^H140R mutant exhibited about 2-fold reduced both ATP affinity (EC_50_ = 39.4 ± 5.0 μM, n = 10) (Fig. 4G,K) and CTP affinity (EC_50_ = 1100 ± 140 μM, n = 9) (Fig. 4H,K) as compared with ^**r**^WT.

The large decrease in the CTP affinity of the ^r^H140A mutant suggested that the hydrogen bond between the basic residue and the cytosine is also conserved in rat P2X4, and is responsible for the affinity for CTP. ^r^H140 is similarly conserved among the P2X1 and P2X4 receptors, primarily as a basic amino residue (Fig. 3E). Furthermore, P2X3 receptors possess a conserved basic residue as the neighbor of ^r^H140 (Fig. 3E). Consistently, CTP functions as a weak affinity agonist for P2X1 (ref. 25), P2X3 (ref. 18) and P2X4 (refs 22 and 26).

### Infrared difference spectroscopy upon the ATP and CTP binding

To further investigate the nucleotide binding specificity of zfP2X4, we applied ligand-binding induced difference Attenuated total reflectance (ATR)-Fourier-transform infrared (FTIR) spectroscopy on the WT and conserved threonine mutants (T189S and T189V) of zfP2X4.

**Figure 5:**
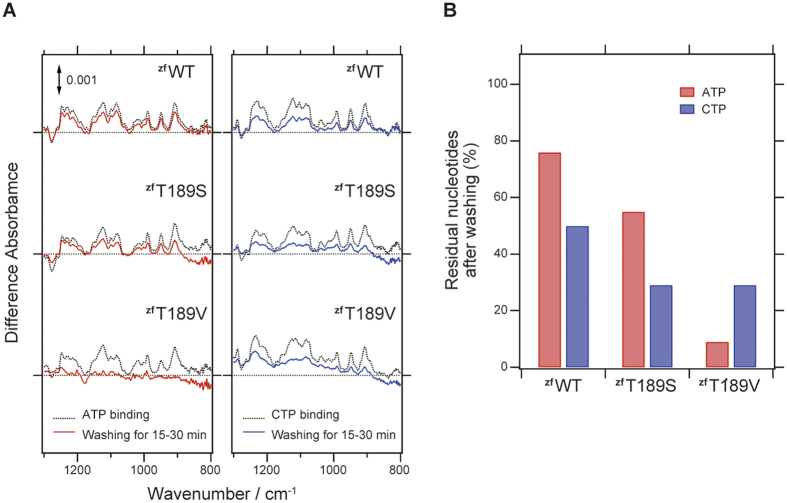
ATR-FTIR spectroscopic analysis of zfP2X4 for the ATP and CTP bindings. (**A**) The reduction of the ligand binding induced difference spectra in the ΔP2X_4_-C ^zf^WT, ^zf^T189S and ^zf^T189V mutants around the P-O stretching region (1250–800 cm^−1^) after washing treatment for 15–30 min. (**B**) The residual nucleotides after the washing treatment estimated from the ATR-FTIR analysis in (**A**).

Figure 5 and Supplementary Fig. S5 compare the difference infrared spectra upon binding of ATP or CTP to the WT and conserved threonine mutants (T189S and T189V) of zfP2X4. The positive side corresponds to the nucleotide-bound state and the negative side to the unbound state, respectively. Several positive bands observed in the 1250–850-cm^−1^ region would be assigned to the P-O stretching modes of a triphosphates group in ATP or CTP as shown in the previous study for small G-protein RAS with GTP^31^. The P-O stretching bands of ATP and CTP are very similar to each other among the WT and conserved threonine mutants (T189S and T189V) (Supplementary Fig. S5A). The small spectral changes are confirmed in the double difference spectra calculated from the ATP and CTP binding-induced spectra as well (Supplementary Fig. S5B). Therefore, the triphosphate groups of ATP and CTP are similarly accommodated in the agonist binding site of P2X receptors, which is consistent with the crystal structures (Fig. 3). Moreover, the spectra in the P-O stretching region are similar among the WT and conserved threonine mutants (T189S and T189V), which suggest that the agonist binding site of the triphosphate group is not perturbed by these mutations. Together with the results from the electrophysiological analysis (Supplementary Fig. S3), these results might indicate that the ligand binding to P2X receptors is not sufficient for the channel gating.

The nucleotide binding affinity of zfP2X4 was analyzed by reduction of the band intensity after washing treatment (Fig. 5). The reduction of the P-O stretching bands between 1250–800 cm^−1^ was monitored for ATP (Fig. 5A). After 15–30 min washing, the residual ATP binding to the WT, T189S, and T189V are ~80%, ~60%, and 10%, respectively (Fig. 5B), and the values for CTP in the WT, T189S and T189V are ~50%, ~30%, and ~30%, respectively (Fig. 5B). We repeated the same experiments and obtained similar results. Therefore, the data showed that the mutation at T189 had more severe effect on the ATP binding than CTP binding, consistent with the ATP-bound and CTP-bound structures (Fig. 3B,D).

## Discussion

In this study, we determined the crystal structure of zebrafish P2X4 in complex with CTP, a low-affinity pyrimidine agonist, and conducted the structure-based electrophysiological and ATR-FTIR spectroscopic analyses. The previously determined ATP-bound structure indicated the functional importance of the conserved threonine for the nucleotide base specificity of the P2X receptors, and suggested that the difference in the number of hydrogen bonds between each nucleotide base and the P2X receptors (three for ATP, two for CTP) is responsible for the weaker affinity for CTP over ATP^12^.

**Figure 6:**
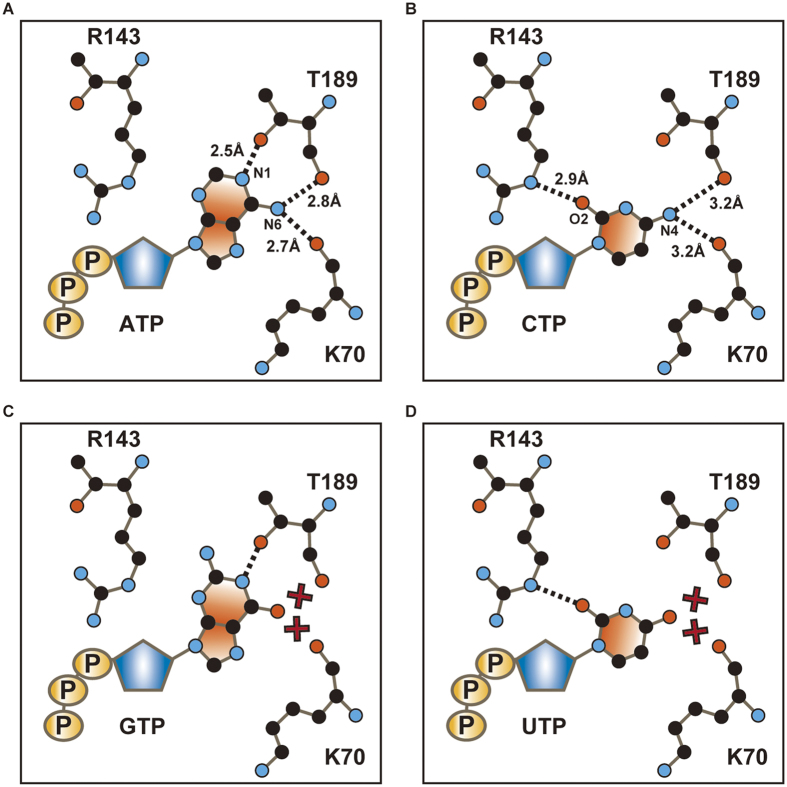
Model for the nucleotide base specificity of P2X receptors. (**A**–**D**) Schematic representations of the interactions between zfP2X4 and ATP (**A**), CTP (**B**), GTP (**C**) or UTP (**D**). Residues involved in nucleotide base recognition and each nucleoside triphosphate are depicted by stick models. Black dashed lines and numbers indicate hydrogen bond interactions, and red crosses indicate non-complementary hydrogen bond partners, despite reasonable hydrogen bonding distances.

Our structural and functional analyses not only verified the functional significance of the conserved threonine at the binding site, but also identified a hydrogen bond between the cytosine base and the basic residue in the agonist binding site, which contributes to CTP binding to the receptor. Accordingly, unlike the previously proposed model, the cytosine base of CTP would be also able to form three hydrogen bonds with the receptor, but possibly with two longer hydrogen bond lengths at the NH_2_ site of CTP (Fig. 6A,B). Therefore, the longer hydrogen bond lengths, rather than the difference in the number of hydrogen bonds, might be mainly responsible for the weaker affinity for CTP over ATP.

Furthermore, the CTP-bound structure also facilitated a reevaluation of the superimposed models of other nucleotide triphosphates onto the agonist binding site (Fig. 6C,D). In the new superimposed models, both GTP and UTP can form only one hydrogen bond between their base rings and the receptor, due to the almost reciprocal hydrogen bonding groups of their base rings, as compared to those of ATP and CTP, respectively (Fig. 6C,D), confirming the explanation for the inability of GTP and UTP to activate P2X receptors.

Overall, our work has revised the previously proposed model for the nucleotide base specificity of P2X receptors, and thus provides new structural insights into the mechanism underlying the nucleotide base specificity. Intriguingly, according to the recent electrophysiological analysis, P2X receptors can be activated by nucleotides other than ATP, such as CTP, at a physiological concentration following an exposure to low concentrations of ATP^21^. Thus, our work might draw more attention to the possible physiological roles of CTP in P2X receptor functions.

Furthermore, P2X receptors have attracted great interest as potential drug targets, and the recent structures of the human P2X3 receptor in complex with the competitive antagonists^14^ and the panda P2X7 receptor in complex with the non-competitive antagonists^15^. In this report, we revealed the mechanism of pyrimidine base recognition by P2X receptors. Intriguingly, some pyrimidine derivative compounds, such as RO-3, work as P2X antagonists^20^, and pyrimidine derivatives targeting P2X receptors may also interact with the receptors in a similar manner. Accordingly, our work might contribute to the further development of pyrimidine derivative antagonists targeting P2X receptors.

## Materials and Methods

### Two-Electrode Voltage Clamp Recordings in *Xenopus laevis* oocytes

The cRNAs encoding zebrafish P2X4 (NP_705939; WT) and rat P2X4 (NP_113782.1; WT and mutants) were transcribed from pGEMHE plasmids^32^, using a mMessage mMachine^®^ T7 ULTRA kit (Ambion, Huntingdon, Cambridgeshire, UK). *Xenopus laevis* oocytes were prepared, and RNA (100 ng for zebrafish P2X4, and 20 ng for rat P2X4) was injected into the oocytes^33^,^34^. The oocytes were incubated at 18 °C in Barth’s solution, containing 88 mM NaCl, 1 mM KCl, 2.4 mM NaHCO_3_, 10 mM HEPES, 0.33 mM Ca(NO_3_)_2_, 0.41 mM CaCl_2_, and 0.82 mM MgSO_4_, pH 7.4, supplemented with 50 μg/ml gentamicin, and were used for recording after 2–3 days. Recording solutions contained 100 mM NaCl, 5 mM HEPES, 2 mM MgCl_2_, pH 7.3, and nucleoside triphosphates (ATP, CTP, GTP, and UTP), which were freshly prepared each day. Oocytes were held at −70 mV with a bath-clamp amplifier (OC-725C, Warner Co., USA), and macroscopic currents were recorded and analyzed using the pClamp 10 software (Molecular Devices, USA)^33^,^34^. We measured n = 4 for zebrafish P2X4 data and n = 7–9 for rat P2X4 data.

### Protein expression and purification

The vector for the expression of the N-terminally GFP-tagged ΔP2X_4_-C was kindly provided by Dr. Eric Gouaux (Oregon Health and Science University). The protein expression, purification and following crystallization were performed as described previously^12^. In brief, we used the same construct, ΔP2X4-C (ΔN27/ΔC24/N78K/N187R), that yielded the ATP-bound crystals. This construct was expressed in Sf9 insect cells as an N-terminal EGFP fusion, with an octa-histidine affinity tag and a thrombin cleavage site between the ΔP2X_4_-C and EGFP. For crystallization, 1 mM ATP and 3 mM CTP were added to the purified ΔP2X_4_-C protein (final 2 mg/ml), to obtain the ATP-bound and the CTP-bound ΔP2X_4_-C crystals, respectively. The ATP-bound ΔP2X_4_-C crystals were used as nuclei for cross-seeding to obtain the CTP-bound ΔP2X_4_-C crystals.

### Protein crystallization

The ATP-bound ΔP2X_4_-C crystals were grown at 4 °C in 3–4 days by the vapor diffusion method, by mixing 1:1, 2:1, or 1:2 (v/v) ratios of protein and reservoir solutions, as described previously^12^. The “1st-generation” CTP-bound ΔP2X_4_-C crystals were grown at 4 °C in 3–4 days, in mixtures of 1:1, 2:1, or 1:2 (v/v) ratios of protein and reservoir solutions, containing 20–26% PEG 2000, 300 mM Mg(NO_3_)_2_, and 100 mM Tris, pH 8.0, by the cross-seeding method using the ATP-bound ΔP2X_4_-C crystals as nuclei. The “2nd-generation” CTP-bound ΔP2X_4_-C crystals were grown under the same conditions, by the cross-seeding method using the “1st-generation” CTP-bound ΔP2X_4_-C crystals as nuclei. The “2nd-generation” CTP-bound ΔP2X_4_-C crystals were harvested and cryoprotected, in a solution containing 25% PEG 2000, 300 mM Mg(NO)_3_, 100 mM Tris, pH 8.0, 25% glycerol, 0.5 mM DDM (n-dodecyl-β-D-maltoside) and 3 mM CTP. Crystals were flash-frozen in liquid nitrogen for X-ray diffraction experiments.

### Data collection and structure determination

X-ray diffraction data were collected at 100 K on the SPring-8 beamline BL41XU (Hyogo, Japan). Diffraction data were processed using HKL2000 (HKL Research Inc.). The structure of CTP-bound ΔP2X_4_-C was obtained by molecular replacement with Phaser^35^, using the ATP-bound ΔP2X_4_-C structure (PDB code 4DW1) as the template. The determined structure was further refined by using the programs PHENIX^36^ and COOT^37^. Crystallographic data and refinement statistics are presented in the table (Supplementary Table S1). All figures were prepared with the CueMol software (http://www.cuemol.org).

### ATR-FTIR measurement

The perfusion-induced difference FTIR spectroscopy was performed as similarly to the previous studies^38^,^39^,^40^. Each zfP2X4 protein was reconstituted into liposome at a protein:lipid (POPC) ratio of 1:100 (mol/mol) in the presence of buffer A (150 mM NaCl, 20 mM HEPES, pH7.0). After exchanging the buffer to 2 mM NaHPO_4_, 10 mM NaCl, pH7.5, the reconstituted liposomes containing ~6 μg of the purified zfP2X4 protein were placed on a diamond ATR crystal (Smith Detection, DurasamplIR II, nine effective internal reflections with an incident angle of 45°). After drying in a gentle stream of N_2_, the sample was filled with buffer A. Before obtaining the spectrum, the sample was washed with buffer A at a flow rate of 1 ml/min for ~1 h. ATR-FTIR spectra of the samples were recorded at 25 °C and 2 cm^−1^ resolution using a VERTEX 70 spectrometer (Bruker Optics) equipped with a liquid-nitrogen-cooled MCT detector. A spectrum of the sample in buffer A was recorded, and the buffer was then switched to buffer A containing 10 μM ATP or CTP, and after washing for 15 min to reach equilibrium, the spectrum of the sample in the presence of the nucleotides was recorded. For each spectrum, 768 interferograms were collected. The obtained difference spectra were normalized based on the absolute absorbance of the amide I band at 1653 cm^−1^, which well correlates with the quantities of the proteins on the ATR crystals. The absolute absorption spectra of the zfP2X4 proteins immersed in buffer A were obtained by subtracting the buffer contribution. After measurement of the absorption spectra before and after the buffer exchange, the difference spectra were calculated by subtracting the two spectra and then correcting for changes due to membrane swelling and non-specific distortions associated with addition of the ligands.

## Additional Information

**Accession codes**: The atomic coordinates and structure factors have been deposited in the Protein Data Bank (http://www.pdb.org), with the accession code 5WZY.

**How to cite this article:** Kasuya, G. *et al*. Structural insights into the nucleotide base specificity of P2X receptors. *Sci. Rep.* **7**, 45208; doi: 10.1038/srep45208 (2017).

**Publisher's note:** Springer Nature remains neutral with regard to jurisdictional claims in published maps and institutional affiliations.

## Supplementary Material

Supplementary Figures and Tables

## References

[b1] Burnstock G. Purinergic nerves. Pharmacol. Rev. 3, 509–581 (1972). 4404211

[b2] Burnstock G. & Kennedy C. Is there a basis for distinguishing two types of P 2-purinoceptor? Gen. Pharmacol. Vasc. Syst. 16, (1985). 10.1016/0306-3623(85)90001-1 2996968

[b3] Brake A., Wagenbach M. & Julius D. New structural motif for ligand-gated ion channels defined by an ionotropic ATP receptor. Nature 371, 519–523 (1994). 7523952 10.1038/371519a0

[b4] Chen C., Akopian A. & Sivilottit L. A P2X purinoceptor expressed by a subset of sensory neurons. Nature 377, 428–31 (1995). 7566119 10.1038/377428a0

[b5] Valera S. . A new class of ligand-gated ion channel defined by P2x receptor for extracellular ATP. Nature 371, 516–519 (1994). 7523951 10.1038/371516a0

[b6] North R. A. Molecular physiology of P2X receptors. Physiol. Rev. 82, 1013–67 (2002). 12270951 10.1152/physrev.00015.2002

[b7] Surprenant A. & North R. A. Signaling at purinergic P2X receptors. Annu. Rev. Physiol. 71, 333–59 (2009). 18851707 10.1146/annurev.physiol.70.113006.100630

[b8] Burnstock G. Pathophysiology and therapeutic potential of purinergic signaling. Pharmacol. Rev. 58, 58–86 (2006). 16507883 10.1124/pr.58.1.5

[b9] Burnstock G. & Ralevic V. Purinergic signaling and blood vessels in health and disease. Pharmacol. Rev. 66, 102–92 (2014). 24335194 10.1124/pr.113.008029

[b10] Gever J. R., Cockayne D. a., Dillon M. P., Burnstock G. & Ford A. P. D. W. Pharmacology of P2X channels. Pflugers Arch. 452, 513–37 (2006). 16649055 10.1007/s00424-006-0070-9

[b11] Kawate T., Michel J. C., Birdsong W. T. & Gouaux E. Crystal structure of the ATP-gated P2X(4) ion channel in the closed state. Nature 460, 592–598 (2009). 19641588 10.1038/nature08198 PMC2720809

[b12] Hattori M. & Gouaux E. Molecular mechanism of ATP binding and ion channel activation in P2X receptors. Nature 485, 207–12 (2012). 22535247 10.1038/nature11010 PMC3391165

[b13] Kasuya G. . Structural Insights into Divalent Cation Modulations of ATP-Gated P2X Receptor Channels. Cell Rep. 14, 932–944 (2016). 26804916 10.1016/j.celrep.2015.12.087

[b14] Mansoor S. E. . X-ray structures define human P2X3 receptor gating cycle and antagonist action. Nature 538, 66–71 (2016). 27626375 10.1038/nature19367 PMC5161641

[b15] Karasawa A. & Kawate T. Structural basis for subtype-specific inhibition of the P2X7 receptor. Elife 5, e22153 (2016). 27935479 10.7554/eLife.22153 PMC5176352

[b16] Haines W. R., Torres G. E., Voigt M. M. & Egan T. M. Properties of the novel ATP-gated ionotropic receptor composed of the P2X(1) and P2X(5) isoforms. Mol. Pharmacol. 56, 720–7 (1999). 10496954

[b17] King B. & Wildman S. Effects of extracellular pH on agonism and antagonism at a recombinant P2X2 receptor. Br. J. Pharmacol. 121, 1445–1453 (1997). 9257926 10.1038/sj.bjp.0701286 PMC1564844

[b18] Garcia-Guzman M., Stühmer W. & Soto F. Molecular characterization and pharmacological properties of the human P2X3 purinoceptor. Mol. Brain Res. 47, 59–66 (1997). 9221902 10.1016/s0169-328x(97)00036-3

[b19] Williams M. & Jarvis M. F. Purinergic and pyrimidinergic receptors as potential drug targets. Biochem. Pharmacol. 59, 1173–85 (2000). 10736418 10.1016/s0006-2952(99)00341-x

[b20] North R. A. & Jarvis M. F. P2X receptors as drug targets. Mol. Pharmacol. 83, 759–69 (2013). 23253448 10.1124/mol.112.083758 PMC3608433

[b21] Browne L. E. & North R. A. P2X receptor intermediate activation states have altered nucleotide selectivity. J. Neurosci. 33, 14801–8 (2013). 24027280 10.1523/JNEUROSCI.2022-13.2013 PMC3771025

[b22] Garcia-Guzman M., Soto F., Gomez-Hernandez J. M., Lund P. E. & Stühmer W. Characterization of recombinant human P2X4 receptor reveals pharmacological differences to the rat homologue. Mol. Pharmacol. 51, 109–18 (1997). 9016352 10.1124/mol.51.1.109

[b23] Lewis C. J. & Evans R. J. Lack of run-down of smooth muscle P2X receptor currents recorded with the amphotericin permeabilized patch technique, physiological and pharmacological characterization of the properties of mesenteric artery P2X receptor ion channels. Br. J. Pharmacol. 131, 1659–66 (2000). 11139444 10.1038/sj.bjp.0703744 PMC1572503

[b24] Ohta T., Kubota A., Murakami M., Otsuguro K. & Ito S. P2X2 receptors are essential for [Ca^2+^]i increases in response to ATP in cultured rat myenteric neurons. Am. J. Physiol. Gastrointest. Liver Physiol. 289, G935–48 (2005). 15905416 10.1152/ajpgi.00017.2005

[b25] Roberts J. a & Evans R. J. ATP binding at human P2X1 receptors. Contribution of aromatic and basic amino acids revealed using mutagenesis and partial agonists. J. Biol. Chem. 279, 9043–55 (2004). 14699168 10.1074/jbc.M308964200

[b26] Soto F. . P2X4: an ATP-activated ionotropic receptor cloned from rat brain. Proc. Natl. Acad. Sci. USA 93, 3684–8 (1996). 8622997 10.1073/pnas.93.8.3684 PMC39672

[b27] Xiong K., Stewart R. R., Weight F. F. & Li C. Role of extracellular histidines in antagonist sensitivity of the rat P2X4 receptor. Neurosci. Lett. 367, 197–200 (2004). 15331152 10.1016/j.neulet.2004.06.008

[b28] Zhang L. . Involvement of Ectodomain Leu 214 in ATP Binding and Channel Desensitization of the P2X4 Receptor. Biochemistry 53, 3012–9 (2014). 24762105 10.1021/bi401711n

[b29] Zhao W.-S. . Relative motions between left flipper and dorsal fin domains favour P2X4 receptor activation. Nat. Commun. 5, 4189 (2014). 24943126 10.1038/ncomms5189

[b30] Tvrdonova V., Rokic M. B., Stojilkovic S. S. & Zemkova H. Identification of Functionally Important Residues of the Rat P2X4 Receptor by Alanine Scanning Mutagenesis of the Dorsal Fin and Left Flipper Domains. PLoS One 9, e112902 (2014). 25398027 10.1371/journal.pone.0112902 PMC4232510

[b31] Xia F., Rudack T., Kötting C., Schlitter J. & Gerwert K. The specific vibrational modes of GTP in solution and bound to Ras: a detailed theoretical analysis by QM/MM simulations. Phys. Chem. Chem. Phys. 13, 21451 (2011). 22048726 10.1039/c1cp22741f

[b32] Liman E., Tytgat J. & Hess P. Subunit stoichiometry of a mammalian K^+^ channel determined by construction of multimeric cDNAs. Neuron 9, 861–871 (1992). 1419000 10.1016/0896-6273(92)90239-a

[b33] Fujiwara Y., Keceli B., Nakajo K. & Kubo Y. Voltage- and [ATP]-dependent gating of the P2X(2) ATP receptor channel. J. Gen. Physiol. 133, 93–109 (2009). 19114637 10.1085/jgp.200810002 PMC2606937

[b34] Hopf T. a. . Amino acid coevolution reveals three-dimensional structure and functional domains of insect odorant receptors. Nat. Commun. 6, 6077 (2015). 25584517 10.1038/ncomms7077 PMC4364406

[b35] McCoy A. J. . Phaser crystallographic software. J. Appl. Crystallogr. 40, 658–674 (2007). 19461840 10.1107/S0021889807021206 PMC2483472

[b36] Adams P. D. . PHENIX: a comprehensive Python-based system for macromolecular structure solution. Acta Crystallogr. D. Biol. Crystallogr. 66, 213–21 (2010). 20124702 10.1107/S0907444909052925 PMC2815670

[b37] Emsley P., Lohkamp B., Scott W. G. & Cowtan K. Features and development of Coot. Acta Crystallogr. D. Biol. Crystallogr. 66, 486–501 (2010). 20383002 10.1107/S0907444910007493 PMC2852313

[b38] Furutani Y., Murata T. & Kandori H. Sodium or Lithium Ion-Binding-Induced Structural Changes in the. J. Am. Chem. Soc. 6, 2860–2863 (2011). 10.1021/ja1116414 21319823

[b39] Furutani Y. . ATR-FTIR spectroscopy revealing the different vibrational modes of the selectivity filter interacting with K^+^ and Na+ in the open and collapsed conformations of the KcsA potassium channel. J. Phys. Chem. Lett. 3, 3806–3810 (2012). 26291114 10.1021/jz301721f

[b40] Furutani Y., Shimizu H., Asai Y., Oiki S. & Kandori H. Specific interactions between alkali metal cations and the KcsA channel studied using ATR-FTIR spectroscopy. Biophys. Physicobiology 12, 37–45 (2015). 10.2142/biophysico.12.0_37 PMC4736833 27493853

