# Supplementary material for: Structural insights into the nucleotide base specificity of P2X receptors
Source: Sci Rep. 2017 Mar 23;7:45208. doi: 10.1038/srep45208 (PMC5362899; doi:10.1038/srep45208)
Supplement: Supplementary Figures and Tables [file srep45208-s1.pdf]

# Supplementary Information

## Structural insights into the nucleotide base specificity of P2X receptors

Go Kasuya[1]*, Yuichiro Fujiwara[2]*, Hisao Tsukamoto[3]*, Satoshi Morinaga[4], Satoshi Ryu[4], Kazushige Touhara[4], Ryuichiro Ishitani[1], Yuji Furutani[3], Motoyuki Hattori[5] & Osamu Nureki[1]

[1] Department of Biological Sciences, Graduate School of Science, The University of Tokyo, 2-11-16 Yayoi, Bunkyo-ku, Tokyo 113-0032, Japan.

[2] Integrative Physiology, Department of Physiology, Graduate School of Medicine, Osaka University, 2-2 Yamada-oka, Suita, Osaka 565-0871, Japan.

[3] Institute for Molecular Science, 38 Nishigo-Naka, Myodaiji, Okazaki 444-8585, Japan.

[4] Department of Applied Biological Chemistry, Graduate School of Agricultural and Life Sciences, The University of Tokyo, 1-1-1 Yayoi, Bunkyo-ku, Tokyo 113-8657, Japan.

[5] State Key Laboratory of Genetic Engineering, Collaborative Innovation Center of Genetics and Development, Department of Physiology and Biophysics, School of Life Sciences, Fudan University, 2005 Songhu Road, Yangpu District, Shanghai 200438, China.

*These authors contributed equally to this work.

Correspondence and requests for materials should be addressed to M.H. (email: hattorim@fudan.edu.cn) or O.N. (email: nureki@bs.s.u-tokyo.ac.jp)

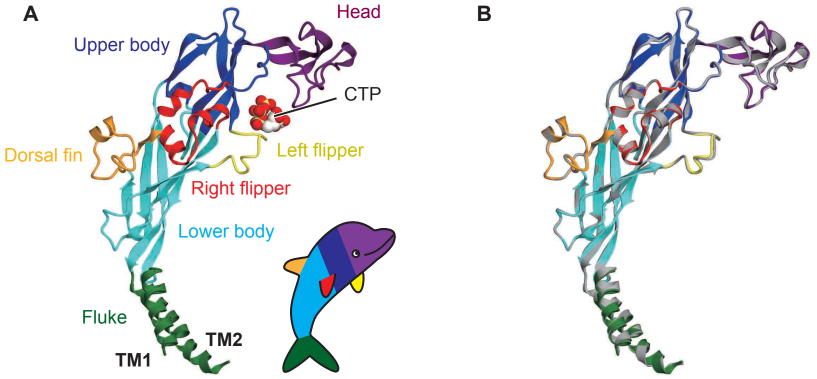


**Figure S1. Subunit fold of the CTP-bound ΔP2X$_4$-C structure.**

(**A**) The CTP-bound ΔP2X$_4$-C subunit, colored according to the dolphin-like model. (**B**) Superimposition of the CTP-bound (colored) and the ATP-bound ΔP2X$_4$-C (grey) structures, using the Cα atoms of the protomers.

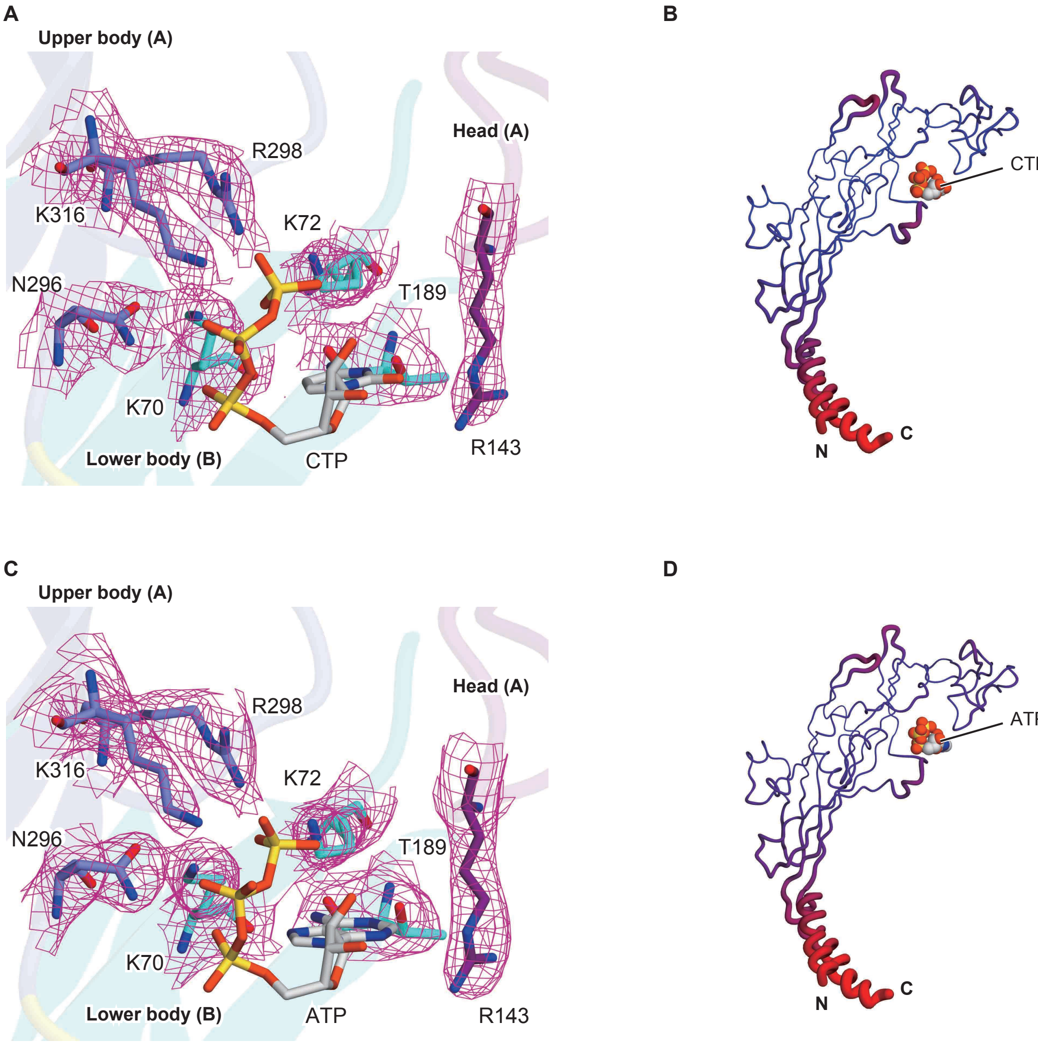


**Figure S2. The electron density maps of nucleotide surrounding residues and the atomic displacement parameter distribution for the CTP-bound and the ATP-bound ΔP2X$_4$-C structures.**

(**A,C**) The $2F_o - F_c$ map contoured at 1.0σ, showing the electron density of the nucleotide surrounding residues in the CTP-bound structure (**A**) and in the ATP-bound structure (**C**). Nucleotides and amino acid residues are depicted by stick models. (**B,D**) The distributions of crystallographic B-factors in the CTP-bound structure (**B**) and in the ATP-bound structure (**D**). The low B-factors are represented in blue while the high B-factors are in red. The thinner tubes mean the lower B-factors (average B-factor for protein is 83.4 Å$^2$ for CTP-bound structure and 89.8 Å$^2$ for ATP-bound structure, with value ranges of 40.5-193.2 Å$^2$ and 46.3-205.9 Å$^2$ respectively).

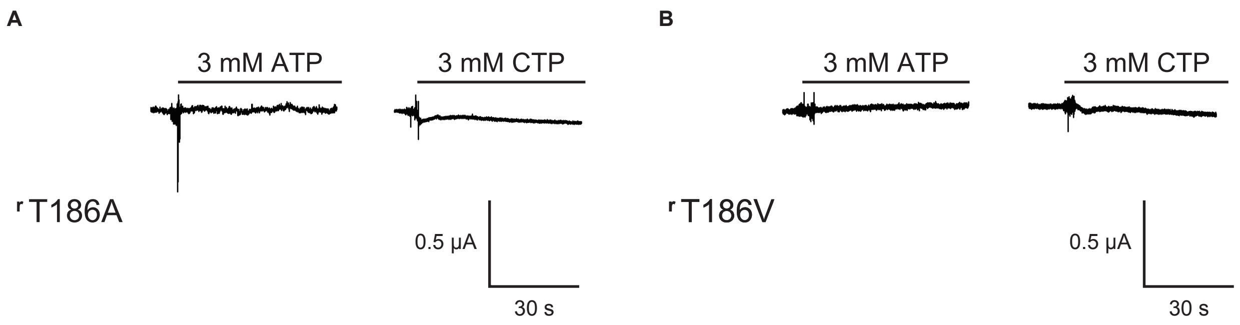


**Figure S3. Current responses of the $^{r}$T186A mutant and the $^{r}$T186V mutant evoked by ATP and CTP.**

(**A,B**) Representative currents of the $^{r}$T186A mutant (**A**) and the $^{r}$T186V mutant (**B**) with the application of 3 mM ATP and 3 mM CTP, showing no evoked currents.

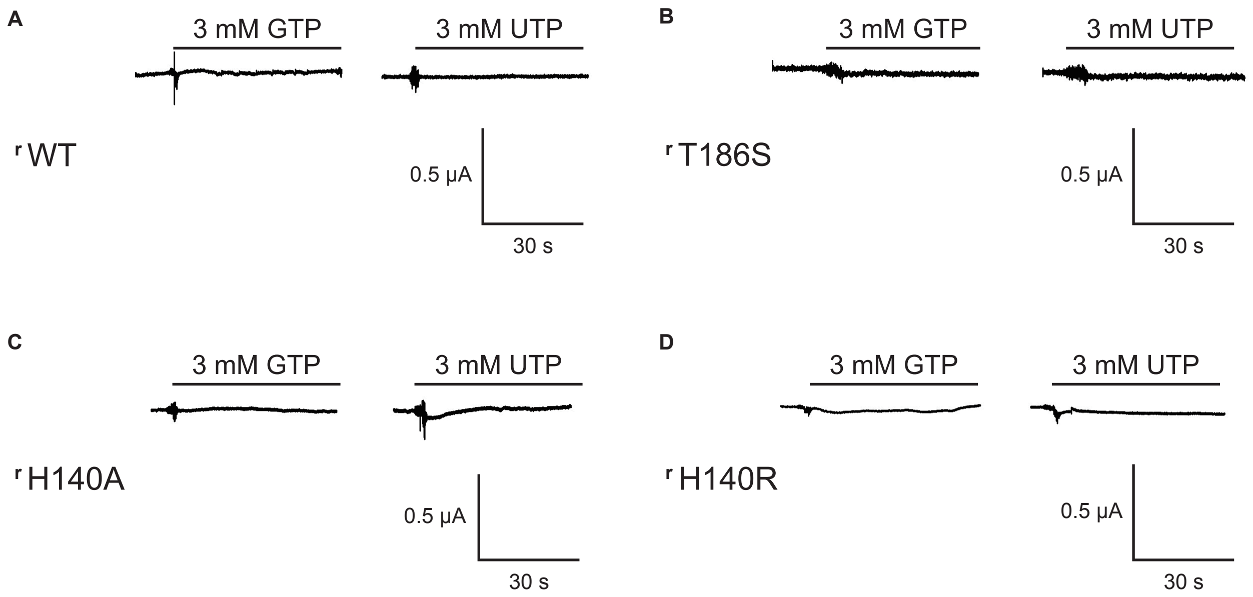


**Figure S4. Current responses of the rWT, the rT186S mutant, the rH140A mutant and the rH140R mutant evoked by GTP and UTP.**

(**A-D**) Representative currents of the rWT (**A**), the rT186S mutant (**B**), the rH140A mutant (**C**) and the rH140R mutant (**D**) with the application of 3 mM GTP and 3 mM UTP, showing no evoked currents.

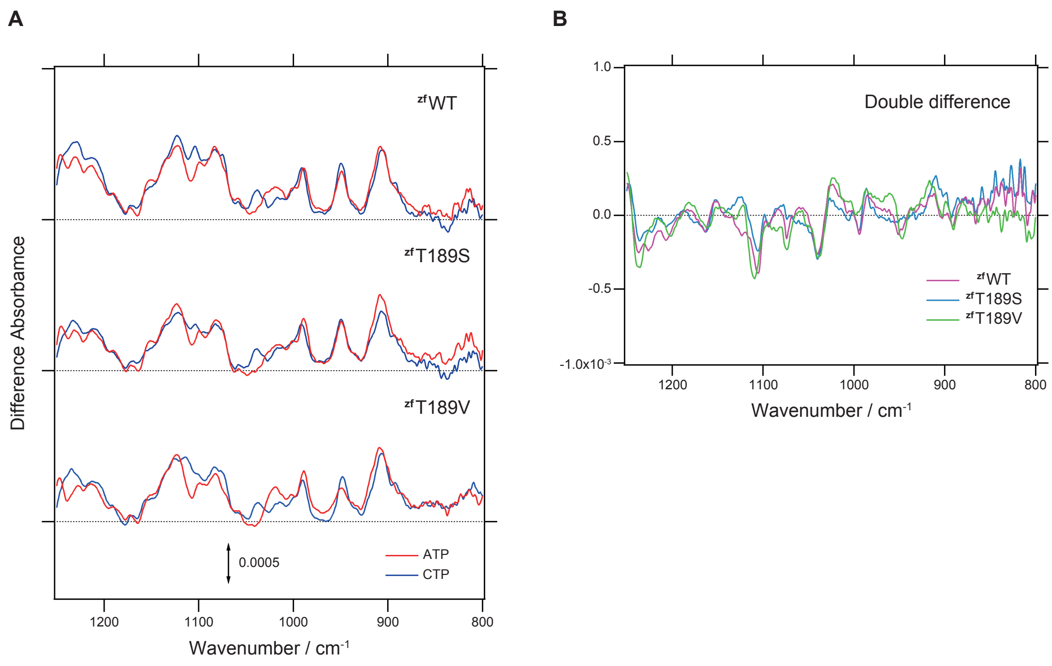


**Figure S5. The ligand-binding induced difference spectra and the double difference spectra of the $^{zf}$WT, the $^{zf}$T189S mutant and the $^{zf}$T189V mutant.**

(**A**) The ligand-binding induced difference spectra in the ΔP2X$_4$-C $^{zf}$WT, $^{zf}$T189S and $^{zf}$T189V mutants upon ATP (red) or CTP (blue) binding (1250-800 cm$^{-1}$). The spectra were obtained by subtracting infrared absorption spectra recorded in the nucleotides free buffer from those recorded in the presence of ATP or CTP. The zero levels are shown by dotted lines. (**B**) The double difference spectra of the ΔP2X$_4$-C $^{zf}$WT (magenta), $^{zf}$T189S (pale blue) and $^{zf}$T189V (green) mutants calculated between the ligand-binding induced difference spectra for ATP and CTP binding of (A).

**Table S1. Data collection and refinement statistics**

| | CTP-bound[a] |
|---|---|
| **Data collection** | |
| Space group | *R32* |
| Cell dimensions | |
| $a$ , $c$, (Å) | 129.2, 252.0 |
| Wavelength (Å) | 1.000 |
| Resolution (Å) * | 50-2.8(2.85-2.80) |
| $R_{merge}$* | 0.119 (0.983) |
| $I$ / s$I$* | 9.4 (1.5) |
| Completeness (%)* | 99. 8[b] (98.8) |
| Redundancy* | 7.8 (5.2) |
| | |
| **Refinement** | |
| Resolution (Å) | 2.8 |
| No. reflections | 20230 |
| $R_{work}$ / $R_{free}$ | 0.212/0.246 |
| No. atoms | |
| Total | 2652 |
| CTP | 29 |
| Water | 72 |
| Average $B$-factor (Å$^2$) | |
| Total | 78.3 |
| Protein | 83.4 |
| CTP | 56.8 |
| Water | 64.1 |
| R.m.s. deviations | |
| Bond lengths (Å) | 0.010 |
| Bond angles (°) | 1607 |
| Ramachandran | |
| Most favored (%) | 90.7 |
| Allowed (%) | 9.3 |
| Generously allowed (%) | 0 |
| Disallowed (%) | 0 |

*Highest resolution shell is shown in parentheses.

[a]ΔzfP2X4-C (ΔN27/ΔC24/N78K/N187R)

[b]9.9% of the reflections were excluded from the refinement for $R_{free}$ calculation.
